# Differentiation without Distancing. Explaining Bi-Polarization of Opinions without Negative Influence

**DOI:** 10.1371/journal.pone.0074516

**Published:** 2013-11-27

**Authors:** Michael Mäs, Andreas Flache

**Affiliations:** 1 Chair of Sociology, in particular Modeling and Simulation, ETH Zurich, Zurich, Switzerland; 2 Department of Sociology/ICS, University of Groningen, Groningen, The Netherlands; University of Maribor, Slovenia

## Abstract

Explanations of opinion bi-polarization hinge on the assumption of negative influence, individuals’ striving to amplify differences to disliked others. However, empirical evidence for negative influence is inconclusive, which motivated us to search for an alternative explanation. Here, we demonstrate that bi-polarization can be explained without negative influence, drawing on theories that emphasize the communication of arguments as central mechanism of influence. Due to homophily, actors interact mainly with others whose arguments will intensify existing tendencies for or against the issue at stake. We develop an agent-based model of this theory and compare its implications to those of existing social-influence models, deriving testable hypotheses about the conditions of bi-polarization. Hypotheses were tested with a group-discussion experiment (*N* = 96). Results demonstrate that argument exchange can entail bi-polarization even when there is no negative influence.

## Introduction

Theories of social and cultural differentiation [1], [2], societal stratification [3], ingroup favoritism [4], political polarization [5], outgroup discrimination [6], and intergroup conflict [7], [8] rely on the assumption that individuals seek to accentuate differences between themselves and negatively evaluated others. Sociological approaches to social differentiation, for instance, explain the development of elaborated cultural norms by the desire of high-status actors to distinguish themselves from individuals with a lower status [1], [3], [9]–[11]. In the same line of reasoning, psychological theories relying on the self-categorization paradigm [8], [12], [13] hold that humans adjust their opinions and behavior in order to minimize the heterogeneity within their ingroup and to maximize differences to outgroups [14].

The notion that individuals seek to intensify differences to disliked others is of particular importance for explanations of the phenomenon of *bi-polarization*, which is defined as the development of increasingly antagonistic groups in a population, where opinion differences between groups intensify and positions between the two extremes of an opinion spectrum are over time increasingly sparsely occupied [15], [16]. Bi-polarization of opinions has puzzled researchers for decades particularly when opinions vary continuously, such as the degree to which voters are in favor or against a political agenda [17], [18]. Building on classical notions of social influence [19]–[21], early formal models of social-influence dynamics in networks [17], [22]–[25] assumed exclusively positive influence. That is, individuals were assumed to always seek consensus with those they interact with, adapting their opinions towards the opinions of their network partners in the course of interaction. Strikingly, these models imply convergence cascades, which eventually drive populations towards overall consensus as long as there are no subgroups that are entirely cut off from outside influences. But, as Abelson [17] observed, the empirical pattern found in extensive research on opinion formation at the community level resembled more that of bi-polarization than of emergent consensus, leaving him to wonder “what on earth one must assume in order to generate the bimodal outcome of community cleavage studies”. Echoing this question, Bonacich and Lu [26] recently included explaining “how groups become polarized or how two groups can become more and more different” in their list of important unsolved problems of sociology.

In search for processes that give rise to bi-polarization despite social influence, an increasing number of models have therefore been proposed that combine both positive influence from similar and negative influence, or distancing, from dissimilar sources [2], [5], [27]–[31]. These models are able to explain bi-polarization in populations where sufficiently many pairs of individuals experience negative social relationships (disliking of dissimilar others). Those individuals further intensify initial opinion differences, gradually developing opposing opinions and, in turn, influencing also initially moderate individuals to adopt opinions on one of the poles of the opinion scale.

Yet, empirical research on negative influence provided mixed evidence and has, in addition, been criticized on methodological grounds [32]. This raises the question whether bi-polarization of continuous opinions can be explained without negative influence. In other words, is it possible that distributions of continuous opinions bi-polarize even in settings where individuals do *not* seek to increase disagreement with negatively evaluated other members of the population? In this paper, we demonstrate that it is possible, analyzing a new theory of bi-polarization, called “Argument-communication theory of bi-polarization” (ACTB). To this end, we develop a formal model of ACTB and report results from computer simulations. The simulations demonstrate that ACTB is able to explain bi-polarization in continuous opinions even in the absence of negative influence and identify the conditions under which ACTB predicts bi-polarization. We then report results of a laboratory experiment that we conducted to empirically test ACTB.

To be sure, we do not argue that theories that assume negative influence fail to explain the bi-polarization dynamics, which have been observed in empirical studies [33]–[35]. Instead, the weak empirical support for the micro-level assumption of negative influence leads us to explore whether and under what conditions it is possible to explain the macro-level phenomenon of bi-polarization without resorting to this assumption. Thus, ACTB offers an alternative explanation of bi-polarization in continuous opinions. The core difference to the existing literature is that ACTB can explain bi-polarization even in settings where individuals are not negatively influenced by others.

The remainder of this article is organized as follows. The next section reviews existing formal models of social-influence dynamics and shows that negative influence is a crucial assumption in existing explanations of bi-polarization when opinions are continuously scaled. We also summarize the outcomes of empirical studies on negative influence. The third section summarizes the core assumptions of ACTB and provides an intuition for why ACTB is able to explain bi-polarization without negative influence. The subsequent section describes the formal model of ACTB and summarizes the results of our simulation study. Next, we describe the empirical study and report the results. In the concluding section, we summarize results and point to future research.

### 1.1. Existing Explanations of Bi-polarization

Bi-polarization tendencies with regard to salient opinions have been documented for example among college students [35] or in culturally diverse work teams [33]. In a similar vein, observers of the dynamics of social and political opinions found tendencies towards bi-polarization on controversial issues such as attitudes towards abortion, sexual morality, and the war in Iraq in the American public, in particular during election periods [34], [36]–[38]. Social interaction in a population does of course not always result in bi-polarization. However, the empirical examples for the phenomenon as well as the potentially severe consequences of bi-polarization in a population render it highly important to understand its mechanisms and conditions.

Classical theories of opinion dynamics in social networks [17], [22]–[25] as well as empirical research [15] on bi-polarization focus on opinions that vary on a continuous scale. In contrast, more recently several models were developed to study dynamics of nominally scaled traits [2], [39]–[45]. These nominal traits represent for instance whether individuals adopt a piece of information [2], [40] or which political party the actors vote for [42].

For the purpose of our study, however, nominal opinion scales are not useful for two reasons. First, many traits do not vary qualitatively and are therefore described more accurately with continuous scales. For instance, very few people agree or disagree exactly with the program of a political party. Instead, people usually agree to a certain degree. Second and most importantly, nominal scales assume that sets of actors hold either perfectly similar or perfectly dissimilar opinions on a given issue. As a consequence, this scale type fails to capture a crucial aspect of empirical research on opinion polarization, the intensification of opinion disagreement on a given issue [15], [16], [34]. We therefore follow classical models as well as empirical research and focus on continuous opinions.

To be sure, continuous opinion scales are a conservative assumption for models that are supposed to explain bi-polarization without assuming negative influence. Previous work has shown that for such models persistent diversity of opinions is particularly difficult to explain when opinions are scaled continuously rather than nominally. Intuitively, the reason is that with continuous opinion scales, even very small opinion similarity is sufficient to trigger a cascade of initially small opinion changes, which gradually decrease opinion differences and eventually result in consensus [46], [47]. With nominal scales, such cascades can only arise across multiple issues, but never within one-dimensional issue spaces as they are investigated in empirical research. In a nominal opinion space, agreement may increase when the number of issues actors agree upon increases gradually, but on any single issue actors are either perfectly similar or perfectly dissimilar [39].

#### 1.1.1. Social segmentation, and homophily

**Figure 1:**
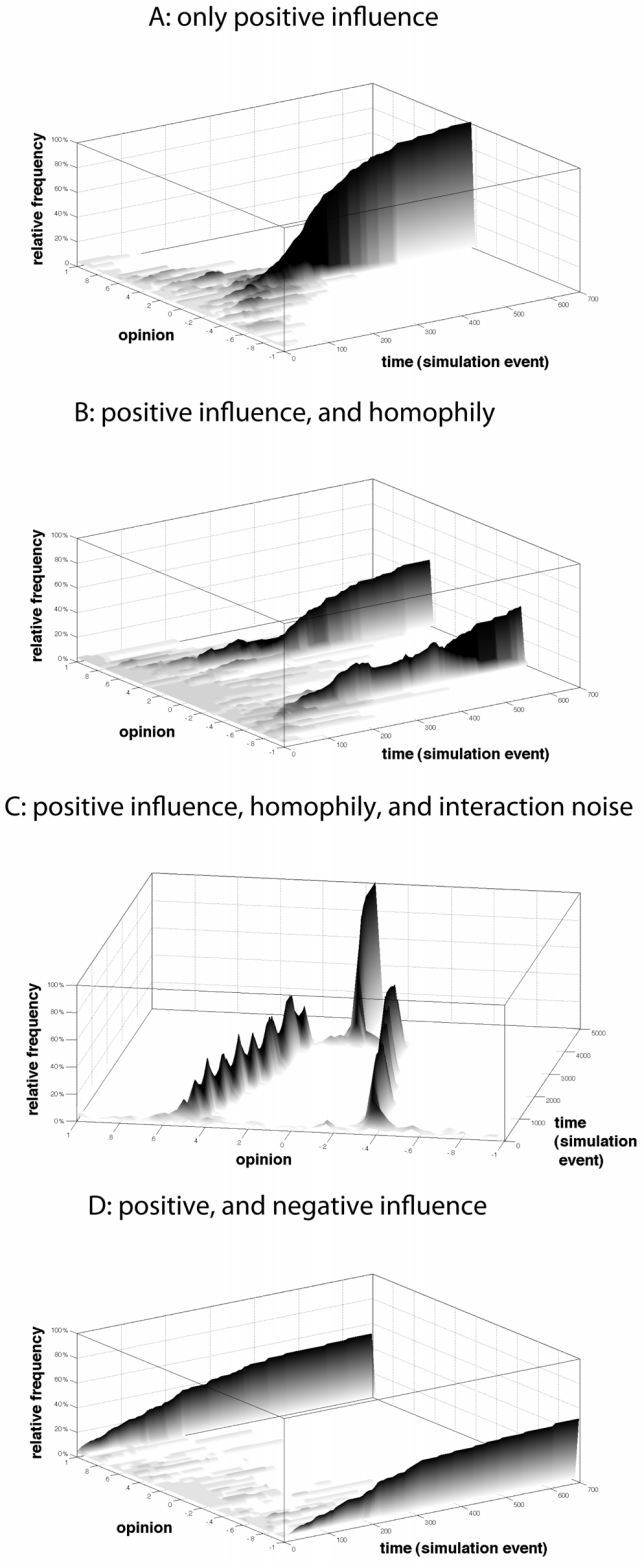
Ideal-typical dynamics generated by existing models of social influence.

Theoretical models of continuous opinion dynamics are based on the assumption of positive social influence [17], [22]–[25]. Individuals adjust their opinions in such a way as to become more similar to their interaction partners, a process which is typically operationalized as opinion averaging [48]. That is, it is assumed that individuals adopt opinions that are equal to the average of their own view and the opinion of influential network neighbors. The surface graph shown in Panel A of Figure 1 provides a typical example of the dynamics that positive social influence generates in a population in which individuals are open to influence from all others. The formal models that we used to generate the graphs of Figure 1 are described in detail in Supporting Information S1. The shading of the surface’s areas and the respective value on the z-axis indicate the relative frequency of individuals that hold a certain opinion at a given point in time. White areas indicate that nobody holds the respective opinion. The darker the area, the more individuals hold this opinion. At the outset of the influence dynamics the opinion is uniformly distributed. However, the figure shows that the variance of the opinion distribution decreases as a result of social influence until, eventually, all individuals hold the same opinion.

Classical contributions demonstrated that the result shown in Panel A of Figure 1 can be generalized. More precisely, researchers proved analytically that perfect uniformity is unavoidable, unless the population is segmented with absolutely no influence between members of different segments [17], [22]–[25]. In clear contrast to this prediction, empirical studies report high diversity of opinions and increasing opinion conflicts even in small groups where no subgroup can avoid interaction with the remainder of the group [33], [35], findings which cannot be explained by social segmentation.

In search for an explanation of opinion diversity, classical models of social influence have been extended by including the assumption of homophily alongside the earlier assumption of positive social influence [18], [40], [43], [49]. In particular, modelers of continuous opinion dynamics incorporated that individuals have a so-called “bounded confidence” [18], [49] in others who hold very different opinions and, thus, interact only with members of the population whose opinions are sufficiently similar to their own. Illustrating the dynamics that social influence implies when agents’ confidence in others is bounded, Panel B of Figure 1 shows ideal-typical dynamics that obtain when initially the opinion is uniformly distributed and individuals are positively influenced only by those others who hold opinions that differ from their own by no more than 20 percent of the range of the opinion scale. As the figure shows, social influence in tandem with homophily can result in the formation of clusters of individuals with minimal opinion variation within and substantial opinion differences between clusters. What is more, according to the bounded-confidence model, this pattern is a stable outcome when opinion differences between clusters exceed the bounded-confidence threshold (20% of the opinion range in the example).

Homophily is a strong force in social interaction [50]–[53] and needs to be taken into account in models of social influence. However, homophily alone fails to provide an explanation of bi-polarization for two reasons. First, as Panel B of Figure 1 demonstrates, social influence in tandem with homophily generates clusters of actors with opinions that are moderate and not bi-polarized. Likewise, opinion differences between clusters do not intensify but remain unchanged once dynamics have settled. In contrast, bi-polarization means that opinion differences between clusters increase over time.

Second, even though homophily plays a key role in many interactions, it appears not reasonable to assume that homophily is the only criterion that guides selection of interaction partners. However, it has been shown that opinion clustering breaks down when tiny deviations from the homophily mechanism are included [47], [54]. As an illustration, Panel C of Figure 1 shows ideal-typical dynamics that unfold when so called “interaction noise” is added to the bounded-confidence model [47]. To be more precise, these dynamics obtain when – unlike in bounded confidence models – there is a positive but very small probability that individuals are influenced even by those others whose opinions falls outside of their bounded confidence range. In this example, the probability of influence in such a dyad was set to 0.01, where influence occurred just like in the classic social-influence models. This is a minimal change in the model assumption of the original bounded confidence model, but it entails a dramatically different dynamic [47]. Figure 1c shows that our modified bounded confidence model can explain clustering only in the short term. In the long run, small deviations from the bounded-confidence assumption lead to social influence between members of distinct clusters and to gradual opinion convergence.

Modelers have studied homophily also on the level of socio-demographic attributes, including the assumption that similarity on demographic variables motivates interaction and social influence [2], [55]. Socio-demographic characteristics are fixed or change at a very slow rate, creating a cleavage along which opinion differences between demographic groups can evolve and intensify. However, socio-demographic cleavages are weak when homophily is based on multiple demographic characteristics that are not perfectly aligned [56], [57]. Accordingly, social-influence models predict that opinion clusters can form in the short term [2] but will eventually converge when socio-demographic attributes are not perfectly correlated [55].

#### 1.1.2. Negative influence

The fragility of opinion diversity in models with continuous opinions lead researchers to search for processes that give rise to bi-polarization. An increasing number of models have been proposed that combine both positive influence from similar and negative influence from dissimilar sources [2], [5], [27]–[31]. This assumption is typically motivated with notions of social balance [58] and the reduction of cognitive dissonance [59]. Intuitively, the idea is that agreement with a negatively evaluated other creates psychological dissonance that can be resolved by changing the own opinion such that disagreement results (negative influence).

Panel D of Figure 1 shows typical dynamics that a combination of positive and negative influence generates in a population that is characterized by an initially uniform opinion distribution. Populations experience bi-polarization if sufficiently many pairs of individuals experience negative social relationships (disliking of dissimilar others) and therefore tend to further intensify initial opinion differences, gradually developing opposing opinions and motivating also initially moderate individuals to adopt opinions on one of the poles of the opinion scale.

Negative influence can generate the same dynamics even when at the outset of the simulation there are no agents with maximally extreme opinions. If initial opinion variance is sufficiently high, then negative influence will intensify opinions that initially lean towards one of the poles and will lead, eventually, to maximally extreme opinions. This dynamic of increased opinion differences also between extremists cannot be generated by models that assume only positive influence and averaging. Averaging implies that opinions will never leave the range of initial opinions [48], [60].

So far, negative influence appears to be the only social mechanism that is able to explain bi-polarization when opinions are continuous and confidence bounds are imperfect. However, empirical tests have hitherto not provided unequivocal evidence in support of the negative-influence assumption. In laboratory experiments, researchers typically informed participants about the opinions of members of fictitious in- and outgroups and then measured pre-test-post-test opinion shifts [32]. The underlying assumption in this line of work was that ingroup members are seen as positive source of influence, while opinions of outgroup members should exert negative influence. These studies have led to mixed results. Many did *not* find support for negative influence [14], [32], [61]. In addition, research illustrates that individuals may publicly distance themselves from others but their private opinions actually do not shift [62].

Moreover, methodological issues cast doubt on the conclusiveness of those studies that researchers interpreted as support for negative influence [14], [63]–[68]. Krizan and Baron [32] raised a number of issues with regard to experiments that focus on negative influence by members of outgroups. For instance, the authors criticize that oftentimes experimental settings do not explicitly refer to a particular outgroup and, thus, do not specify the point of reference for negative influence [69], making it difficult to attribute observed opinion changes to the motivation to intensify differences to outgroups. In addition, a typical strategy adopted in, e.g., experiments based on the minimal group paradigm [70] is to assign participants to temporary groups, “raising questions regarding whether any significant feelings of group identification develop” [32].

We point here to two major additional problems. First, some experimental designs do not allow to disentangle positive influence from the ingroup and negative influence from the outgroup in the explanation of opinion shifts [14], [67], [68]. In these studies, participants have been exposed two sources of social influence, ingroup members and outgroup members. Participants were exposed to ingroup members who held opinions relatively similar to their own. Some of these ingroup members held more extreme opinions than the participant. Outgroup members always had opinions distinct from those of the participants. With such a design, opinion changes away from the outgroup opinion may have been caused by both negative influence from the outgroup or positive influence from more extreme ingroup members [71].

A second problem is that other studies did not control for general opinion drifts during the experiment [64], [65]. For example, Mazen and Leventhal [64] confronted expectant mothers with a favorable description of breast feeding and measured how this affected the mothers’ opinions on this issue. They found that mothers developed more positive opinions when they received information from a communicator with a similar skin color (positive influence). However, when the communicator and the mother were dissimilar in skin color, the opinions of the mothers turned more negative. This suggests that these mothers were influenced negatively by the communicator. However, this result may also have been caused by a general trend towards more negative opinions. In this study, the second opinion measurement took place one week after the first. In this period, all participants might have developed more negative opinions. Possibly, those mothers who where similar to the communicator were positively influenced by them and changed their minds back to more positive opinions. The opinions of the dissimilar mothers, however, might have been unaffected by the communicator’s information and remained more negative. Such trend effects were not controlled for in these analyses. It is therefore not clear whether the reported opinion dynamics are the result of negative influence or of opinion drifts.

In a nutshell, existing theories of bi-polarization in continuous opinion dynamics critically hinge on the assumption of negative influence. However, there is hitherto no conclusive empirical evidence supporting this assumption. In the following, we propose, analyze, and the test an alternative approach that does not rely on negative influence.

### 1.2. Explaining Bi-polarization without Negative Influence

In the tradition of classic notions like Adam Smith’s famous “invisible hand”, social scientists have emphasized that social phenomena can emerge even though individuals do not strive to create them or actually even seek to prevent their emergence [72], [73]. Most prominently, Schelling [73] demonstrated that residential segregation can emerge even though individuals accept to live in neighborhoods where the vast majority of their neighbors holds different demographic characteristics. Similarly, populations may fail to produce collective goods even though all members of the population have a great interest in the provision of the good, because everyone assumes that collective action will succeed without their own contribution [74].

In a similar vein, we propose that initially homogeneous populations can fall apart into subgroups with opposing opinions even though individuals do not seek to distance themselves from any other member of the population and social influence is only positive. Our new theory, called “Argument-communication theory of bi-polarization” (ACTB), is inspired by earlier theorizing on demographic faultlines [56] and group polarization [75]–[77], which combined insights from Persuasive Argument Theory (PAT) [78], [79] and research on homophily [50], [51], [53], [80], [81].

PAT [78], [79] assumes that individuals base their opinions on pro and con arguments. During discussion, individuals are exposed to the arguments their interaction partners consider relevant. In groups where members tend towards a specific opinion already prior to discussion, mainly those arguments will be brought up that favor the prevailing tendency. Discussion members, thus, provide each other with further arguments that support their initial position. This intensifies opinions and aggregates to a *collective* opinion shift towards more extreme positions.

Building on earlier work [56], [75], [76], we argue that the interplay of the argument communication described by PAT with homophily can give rise to bi-polarization. The idea is that small initial opinion differences in a group are gradually amplified when argument communication occurs more frequently between those individuals who initially have relatively similar opinions than between those whose opinions are relatively dissimilar. Due to homophily, individuals with opinions leaning towards the same pole of the opinion spectrum interact more likely with each other than with those who lean towards opposite poles. Thus, persuasive argument communication reinforces existing opinion tendencies, but in opposing directions in the separate subsets of group members who share the same initial tendency. This further reduces the likelihood of interaction between initially dissimilar pairs of individuals, which in turn further strengthens existing tendencies. This process unfolds simultaneously at both sides of the opinion spectrum, such that a self-reinforcing dynamic may arise that entails bi-polarization even in the absence of negative influence.

The core ingredient that ACTB adds to existing approaches to social influence is the communication of arguments. Abstracting from arguments, existing social-influence models assume that individuals inform each other about their opinions during interaction, a process which modelers typically implemented as opinion averaging [17], [18], [23], [24], [48], [49]. On the one hand, averaging appears to be a realistic operationalization of social influence when individuals with different opinions influence each other, because it implies decreasing opinion differences. On the other hand, averaging implies that individuals do not adjust their opinions when they interact with others with whom they already agree, because the average of two identical values is similar to these values. In clear contrast, social psychological [75] and sociological [82] research on the opinion dynamics in discussion groups suggests that through the communication of arguments interaction partners with similar opinions can provide each other with new arguments which reinforce their initial opinion and, thus, leads to intensified views that may be more extreme than those of any of the participants were prior to the interaction [75], [78].

## Analyses

### 1.3. A Formal Model of Argument Exchange

#### 1.3.1. Purpose of the formal study

Developing a formal representation of ACTB was necessary for two main reasons. First, we sought to formally demonstrate the logical validity of the counter-intuitive claim that bi-polarization can emerge even in the absence of negative influence. Likewise, a formal analysis of the theory was necessary because its ability to explain bi-polarization might hinge on potentially problematic assumptions. For instance, bi-polarization requires homophily according to our informal reasoning. However, it remains unclear how strong homophily needs to be, in order to render bi-polarization a likely outcome of the dynamic. As long as there is some probability of interaction also between actors with dissimilar opinions, bi-polarization tendencies might be very unlikely. When actors with dissimilar opinions interact, they likely exchange arguments that speak against their current tendency and lead to more moderate opinions. Furthermore, in subsequent interaction actors will transmit these counter arguments to similar others. This will lead to further opinion convergence. In sum, this reasoning suggests that even though actors may tend to interact with similar others, occasional deviations from this rule may suffice to impede the bi-polarization tendencies generated by argument communication and homophily. In a non-deterministic world, there is no guarantee that a self-reinforcing dynamic eventually leads a social system into the state towards which the dynamic pushes it. This has for example been demonstrated for formal stochastic models of residential segregation [83], or cultural dissemination [84]. In these models, the “ordered” outcomes towards which individual decision rules drive the system, such as highly segregated residential distributions, or local clustering of similar cultures, only arise when the level of randomness in individual decision making is sufficiently small. Accordingly, we sought to analyze how strong homophily needs to be in order to give rise to bi-polarization.

Second, the formal model of ACTB guided the design of the laboratory experiment. The highly controlled nature of laboratory experiments allowed us to incorporate the core design features of the experiment in our formal model and to study the theoretical implications of argument exchange in this particular setting. These model implications could be compared to predictions of existing models of exclusively positive [17] and simultaneous positive and negative [29] influence, helping to identify the conditions under which ACTB implies predictions distinct from those of existing models. These conditions were implemented in the design of our laboratory experiment, creating a decisive test of the new theory against existing models of bi-polarization.

#### 1.3.2. The formal model

Our agent-based model of ACTB implements the substantive assumptions of PAT and homophily for each of *N* interdependent individuals who simultaneously participate in an artificial influence process. Each individual is represented as an agent *i*, with a numerically valued opinion *o_i,t_* (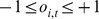) which represents the agent’s stance on a given issue at time point *t*. We assume that there is a limited number of arguments that address the issue. The valence of an argument is expressed numerically. More precisely, *P* pro arguments (*a_l_* = 1) and *C* con arguments (*a_l_* = −1) are available. This is summarized in the argument vector, an array of arguments with *P*+*C* elements. Elements with a row number smaller than *P*+1 hold pro arguments, i.e. *a_l_* = +1. The remaining elements contain con arguments, i.e. *a_l_* = −1.

Empirical research suggests that people have limited capacities to remember and process information [85], [86]. Accordingly, we assume that at a given time point *t* agent *i*’s opinion is based only on a subset of *S_i,t_* relevant arguments (*S_i,t_*≤*P*+*C*). The remaining arguments are not relevant in the opinion formation. For each agent *i*, the relevance vector summarizes which of the arguments are relevant. This vector has *P*+*C* elements which adopt the value one if the respective argument *l* is considered relevant (*r_i,t,l_* = 1) and zero if the argument is not relevant (*r_i,t,l_* = 0).

Technically, an agent’s opinion is the average value of the arguments *a_l_* that the agent considers relevant (see equation 1). For simplicity, we assume that all relevant arguments have the same persuasiveness. This is expressed by the assumption that all relevant arguments are equally weighted in the calculation of the opinion.
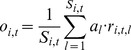
(1)


For example, an agent *i* that bases her opinion on 6 pro arguments (*S_i,t_* = 6) holds an opinion of *o_i,t_ = *1. However, if the agent considers 3 pro and 3 con arguments relevant, then the opinion will adopt the value zero.

Following research on memory processes [87] and existing models of social influence [2], [40], we assume that agents disregard pieces of information that have not been communicated in recent interactions. This is implemented for each agent in a recency vector. This vector has *P*+*C* elements and each element indicates how recent the respective argument is for the agent. Elements of the recency vector with a row number smaller than *P*+1 identify the relevance of pro arguments. The remaining elements determine the relevance of con arguments. Arguments are either relevant or not, but agents rank the *S_i,t_* relevant arguments according to their recency. We denote the recency of an argument (*s_l,i,t_*) with integer values between 0 and *S_i,t_* (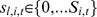). A value of *s_l,i,t_* = 0 indicates that the argument *a_l_* is *not* sufficiently recent and therefore *not* relevant for actor *i*. Values above zero indicate that this argument *is* sufficiently recent and therefore affects actor *i*’s opinion. The most recent argument has the value of *s_l,i,t_* = *S_i,t_,* the second most recent argument has the value *S_i,t_*−1, and so on. Thus, if an agent considers three arguments (*S_i,t_* = 3) then one argument has a recency of 1, one has a recency of 2, and one has a recency of 3. The recency rank of an argument does *not* affect the extent to which an argument shapes the current opinion (see equation 1). However, the recency determines *how long* an argument affects the agent’s opinion in the influence process. The exact rules for updating argument recency will be elaborated further below.

Similar to existing models of social influence [2], [39], [49], we model the opinion formation process as a sequence of events *t*, each event corresponding to one interaction between two agents. An interaction consists of a partner selection phase and a subsequent social influence phase. In the partner selection phase, two agents from the population are matched for interaction, based on opinion-homophily. Subsequently, an opinion of one of the interacting agents is updated as a result of the interaction. The updating rule operationalizes the argument exchange mechanism of PAT.

We implement the *partner selection* phase as follows. In each event, the computer first randomly picks an agent i*. Then an interaction partner *j* (*j*≠i*) is selected. The probability that agent *j* is chosen as interaction partner depends on the similarity between i* and *j*, sim_i*,j,t_, that varies between 0 and 1. A similarity of zero expresses maximal dissimilarity, whereas sim_i*,j,t_ = 1 if both actors hold exactly the same opinion. Formally,
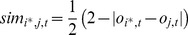
(2)


The probability that agent i* chooses *j* as interaction partner (*p_j,t_*) derives from their relative similarity, that is: the degree to which *j* is more similar to *i* than other group members are. Technically,
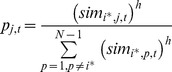
(3)


Equation 3 implements homophily. The more similar *j* is to i* the higher is the probability that they will interact [2], [40]. If two agents hold maximally opposing opinions then the probability of interaction equals zero. Making it possible to vary the *strength of homophily*, we include the parameter *h* into the model. The higher the value of *h*, the steeper is the increase of the likelihood that *j* will be chosen by i* as an interaction partner in the relative similarity of i* and *j*. The actual selection of the interaction partner of i* is implemented by a random draw of one agent from the set of all other group members, based on the probabilities *p_j,t_* given by (3).

Next, i* is socially influenced by the selected interaction partner j* based on the persuasive arguments mechanism. For this, the computer randomly picks one argument, a_l*_, out of the S_j*,t_ arguments that j* considers relevant. Each relevant argument has the same probability to be chosen (1/S_j*,t_). Arguments that are not relevant for j* are not chosen. The chosen argument is then adopted by i*. Technically, its recency for i* is updated to a value of S_i*,t_+1 (s_l*,i*,t_ = S_i*,t_+1). Subsequently, the recency of all arguments that have non-zero recency in i*’s recency vector is reduced by one, if prior to the interaction the corresponding argument was more recent for i* than the argument adopted from j*. As a result, the argument that was communicated by j* becomes relevant for i* and attains the highest recency of all argument that i* considers relevant (s_l*,i*,t_ = S_i*,t_).

This updating procedure implements the assumptions that agents tend to forget dated information [2], [40], [87]. It implies in particular that agents forget one of the arguments previously relevant for them, if they have learned a new argument in the interaction. This assures that the number of arguments that is relevant for an agent is kept constant at *S_i,t_* throughout the influence process. Technically, this assumption makes the subscript *t* in *S_i,t_* superfluous. Below, however, we also consider a model version where agents do not forget arguments because in the setting of the laboratory experiment it was very unlikely that participants forget arguments.

Interaction events are iterated until the system reaches equilibrium. Our model has exactly two equilibria, perfect consensus and maximal bi-polarization. Perfect consensus is reached when all agents hold the same opinion and base it on the same set of arguments. Perfect consensus is a stable situation because agents can transmit only arguments that their interaction partners already consider relevant. This implies that opinions will not be affected by argument exchange. Maximal bi-polarization obtains if there are two maximally distinct subgroups and the members of each subgroup agree on opinions and arguments with each other. That is, the members of the subgroups have coordinated on the opposite poles of the opinion scale and the pairwise similarity (*sim_i,j,t_*) between agents of different subgroups is zero. In this situation, the probability is zero that agents interact who belong to different subgroups (see equation 3). Argument exchange between the subgroups is thus precluded. In addition, interaction of agents that belong to the same subgroup can not lead to opinion changes because these agents base their opinion on either exclusively pro-arguments or exclusively con- arguments. Any outcome of the process that is not perfect consensus or perfect bi-polarization can not be an equilibrium. The reason is that any other outcome implies that there are differences in opinions or arguments between agents, and a positive probability of interaction between the agents who hold different opinion or arguments. There is thus a positive probability that the distribution of arguments and opinions in the population will change due to interaction.

#### 1.3.3. Dynamics of Bi-polarization: an illustrative simulation run

**Figure 2:**
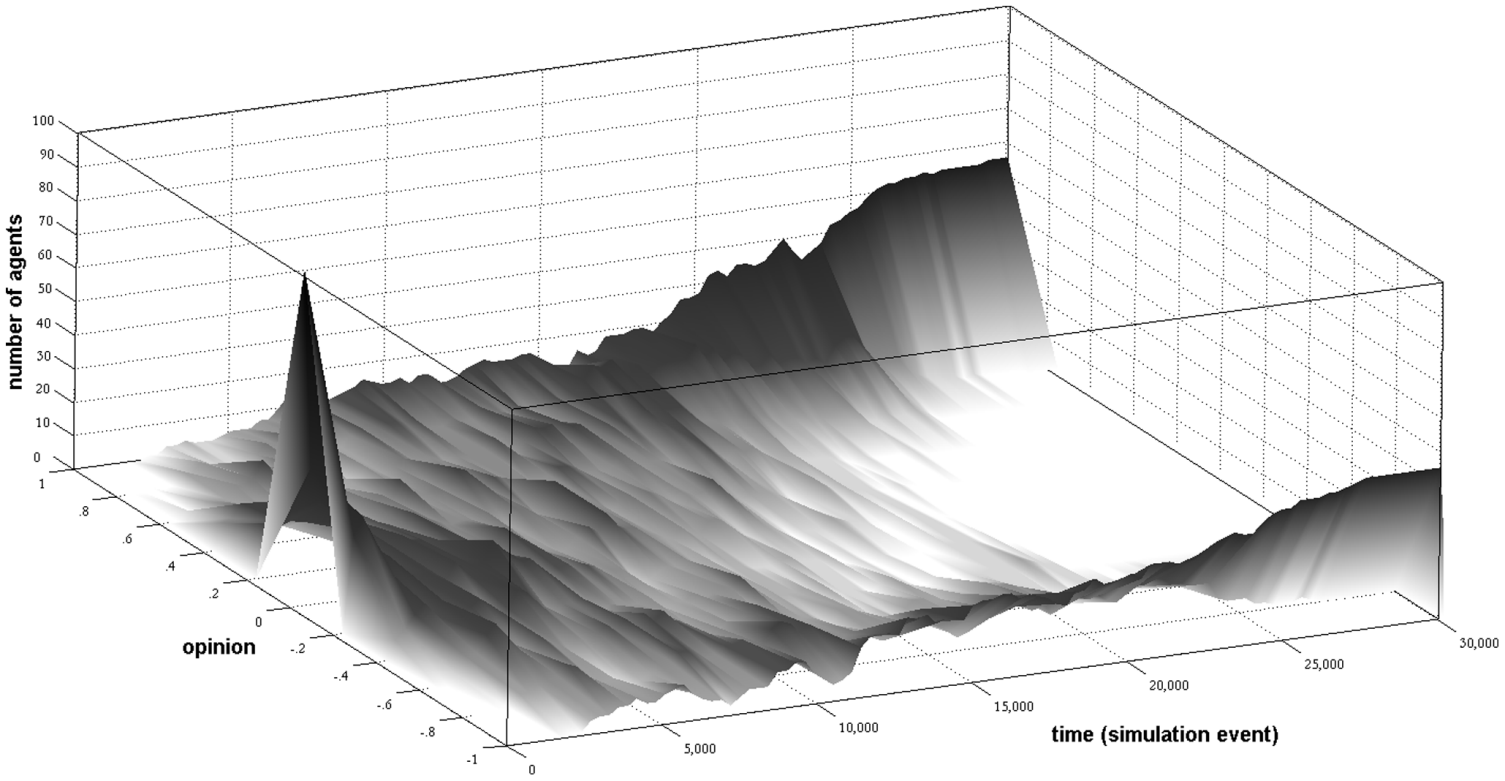
Bi-polarization generated by argument exchange and homophily (*N* = 100, *P* = *C* = 30, *S* = 10, *h* = 9).

Figure 2 shows a surface graph that shows ideal-typical opinion dynamics that the formal model of ACTB generates. For this illustrative simulation run, we imposed conditions for which ACTB predicts bi-polarization tendencies to be very strong. Accordingly, we imposed relatively strong homophily, assuming *h* = 9. With this value, homophily is so strong that interaction between agents who do not hold perfectly similar opinions is extremely unlikely. Furthermore, we assumed that thirty pro and con arguments are available (*P* = *C* = 30) and all agents always consider 10 relevant arguments for opinion formation (*S_i,t_* = 10 for all *i* and *t*). For this condition, we simulated a population of 100 agents, the same population size that we assumed for the ideal-typical simulation runs that are shown in Figure 1. We studied the change of agents’ opinions and argument vectors over 30,000 simulation events. The latter implies that each agent’s opinion is updated 300 times on average.

The initial distribution of arguments and opinions was created by assigning to each agent a random set of 5 pro and 5 con arguments. With this, all agents started with the same opinion at the middle of the opinion scale (*o_i,0_* = 0 for all *i*). Thus, at the outset there were no opinion differences between agents. According to ACTB, this does not rule out opinion changes, as there are still differences between agents in the arguments on which their opinions are based. In contrast, social-influence models that implement influence as opinion averaging predict zero future opinion changes when all agents hold the same opinion [48]. The same holds for models that assume both positive and negative social influence, because in these models negative influence only occurs when there are sufficiently strong opinion differences between agents already at the outset of the influence process [88]. Hence, ACTB and existing models of social influence imply critically different opinion dynamics that unfold from perfect opinion uniformity, making this an interesting initial condition to demonstrate the theoretical implications of ACTB.

Figure 2 shows a surface graph which depicts the development of the opinion distribution during a typical simulation run. At the beginning of the simulation (event zero), all 100 agents hold the same opinion. The figure shows how bi-polarization emerges in this simulation run. While opinions are approximately uniformly distributed after about 10,000 simulation events, the distribution becomes bimodal after about 15,000 events. Subsequently, the two modes gradually become more accentuated and shift towards the opposite ends of the opinion spectrum until, after about 30,000 events, the population is almost entirely split into two approximately equally large subsets of agents with opinions of −1 and +1, respectively.

Opinion change is possible despite initial uniformity, because agents base their opinion on different (randomly assigned) sets of five pro and five con arguments. Thus, in some interactions agents’ opinions shift away from the initial consensus, because they learn a new pro (con) argument and forget a con (pro) argument. Their new opinion is then based on more pro (con) than con (pro) arguments and takes a positive (negative) value. Figure 2 shows that this results in an increase of the variance of the opinion distribution in the first phase of the simulation run. After about 10,000 simulation events, the opinion is approximately uniformly distributed. Due to the strong homophily, agents are matched with interaction partners that have adjusted their opinion in the same direction. These interaction partners will more likely provide each other with arguments that further intensify their opinion tendency rather than to communicate arguments that render their opinions more moderate again. Eventually the opinion trajectories of all agents move to one of the two outer ends of the opinion scale. At this point, the opinion distribution stabilizes, because agents base their opinions on either only pro or only con arguments such that interaction is only possible between agents who already hold identical opinions. Agents can no longer learn arguments that could change their opinions.

#### 1.3.4. Effects of homophily

According to ACTB, communication of persuasive argument can create bi-polarization only if interaction partners are selected based on homophily. In order to test whether homophily always entails bi-polarization, or whether bi-polarization can only arise when homophily is sufficiently strong, we conducted a simulation experiment in which we varied the model parameter *h* between 0 (no homophily) and 8 (strong homophily) in steps of 1. Per condition, we ran 500 independent realizations of the simulation. In all simulations of this experiment, we studied populations of 20 agents (*N* = 20). This is a plausible group size for school classes and work teams [89], two of the settings for which theory and empirical accounts of intra-group conflicts suggest the possibility of bi-polarization dynamics [56].

Research on human cognitive capabilities suggests that humans can process and recall between 4 and 7 chunks of information [85], [86]. For the simulation experiment, we therefore imposed that agents always base their opinions on six arguments (*S_i,t_* = 6 for all *i* and *t*). We replicated the simulation experiment with higher and lower values of the parameter *S* and did not find qualitative differences. We found stronger bi-polarization when agents consider fewer arguments for opinion formation. This effect obtains because it takes agents at least *S* interactions to drop a newly adopted argument. Thus, when an agent with an extreme opinion happens to adopt a counter argument, then this counter argument will remain relevant longer if *S* is high. It follows that this agent will hold a more moderate opinion for a longer period. This, in turn, increases the probability that the agent interacts with agents that hold opposing opinions and adopts further counter arguments. In sum, high values of *S* make it more likely that agents with an extreme opinion adopt moderate opinion values and therefore decrease bi-polarization.

Furthermore, we assumed that there are 20 pro and 20 con arguments (*P* = *C* = 20) available. These values create sufficient variation in the initial argument sets also between agents who happen to hold identical opinions. For this, *P* and *C* should considerably exceed *S*. Otherwise agents with similar opinions are likely to base their opinions on similar sets of arguments. This would preclude the possibility that argument exchange between agents with similar opinions renders their opinions more extreme because they provide each other with arguments that they already consider relevant. Furthermore, we created the initial condition such that opinions are uniformly distributed. For this, we randomly assigned to each agent one of the *S*+1 possible opinion values and then randomly picked one of the possible sets of *S* arguments which correspond to the selected opinion value.

**Figure 3:**
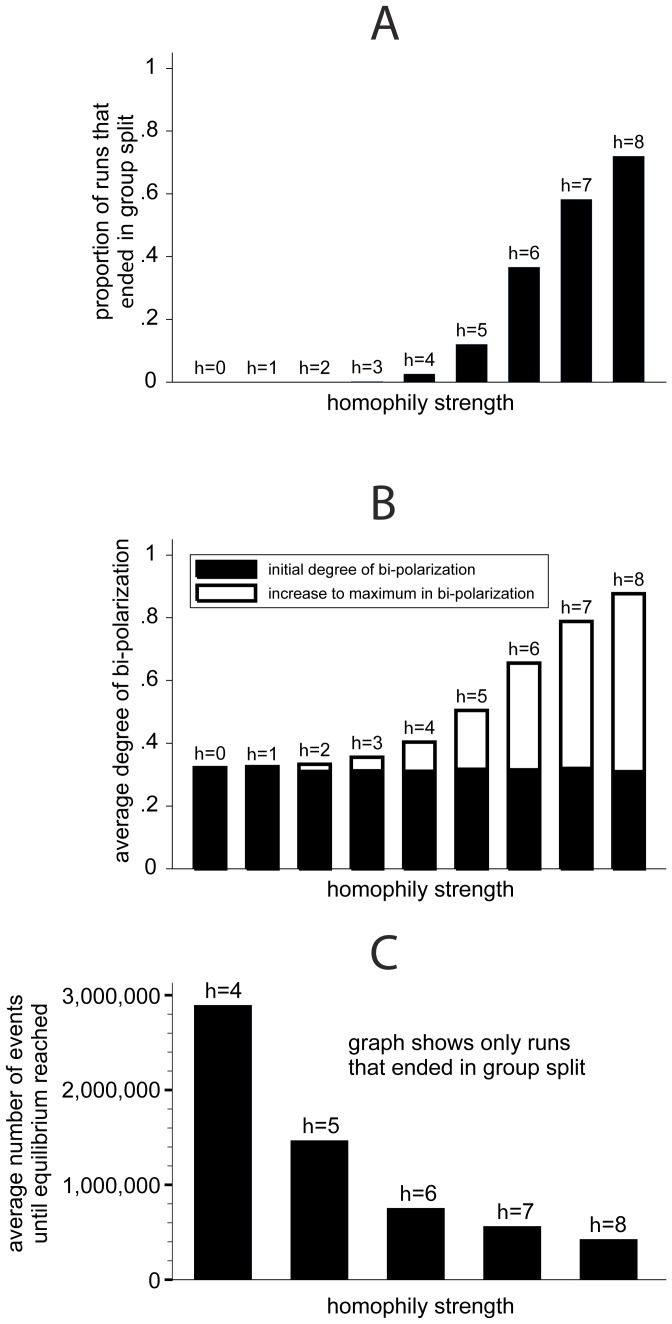
Results from simulation experiment on the effects of homophily on the degree of bi-polarization (500 runs per condition, *N* = 20, *P* = *C* = 20, *S* = 6).

Figure 3 summarizes the results. Panel A of Figure 3 shows how homophily strength *h* affected the proportion of runs that ended in a perfect split into two maximally antagonistic subgroups. When homophily strength was below *h* = 3 all runs ended in consensus. At *h* = 3, only one out of the 500 replications for this condition ended in a group split with two subgroups at the opposing poles. For higher values of homophily strength *h*, panel A shows that the stronger homophily was, the more runs ended in a perfect group split.

If a simulation run ends in perfect consensus, there may nonetheless have been a temporary period of significant bi-polarization in the dynamic. To test for this possibility, we assessed for each event of the simulation runs the degree to which the population was bi-polarized. In the following, we refer to this as the “degree of bi-polarization” at a given point in time. Following Flache and Mäs [28], [90], the degree of bi-polarization was measured with the standard deviation of the distribution of pairwise opinion distances between all pairs of agents in the population. This measure takes its maximal value, one, when there are two equally large and maximally different subgroups. The minimal value, zero, is obtained for perfect opinion consensus. In between these two extremes, the bi-polarization measure increases in the extent to which the opinion distribution is bimodal, with equally large modes at opposite extreme ends of the opinion spectrum. Panel B in Figure 3 compares the average degree of bi-polarization at the outset of the simulation runs with the average maximal degree of bi-polarization that occurred during the simulations. Under all conditions, the simulations started with random, uniform opinion distributions. This resulted for all conditions in a low degree of bi-polarization in the initial situation (indicated by the black areas of the bars). The white areas of the bars indicate the average maximal degree of bi-polarization obtained in a simulation run. Panel B shows that the stronger homophily, *h*, the higher the increase of bi-polarization between the initial condition and the maximum level of bi-polarization. Furthermore, bi-polarization increased only slightly in the course of a simulation run when homophily was weak (*h*<4). In these conditions the simulated populations hardly bi-polarized. Only strong homophily could give rise to significant levels of bi-polarization.

Finally, we wanted to know whether higher homophily strength accelerated the emergence of bi-polarization. For this, panel C in Figure 3 informs about the average number of simulations events that it took to reach the equilibrium in those runs that ended with perfect bi-polarization. This measure serves as an indicator of the duration of the bi-polarization process. The conditions with weak homophily (*h*<3) are neglected in Panel C, because only one out of 2000 runs ended in bi-polarization in these conditions. The graph shows that the weaker homophily the more events it took until bi-polarization was reached. These results indicate that bi-polarization is not only possible under strong homophily. The self-reinforcing process that leads to bi-polarization may evolve also if homophily is only moderately strong. However, with moderate homophily it may take a considerable amount of time until a group splits up into opposing factions. This also highlights the mechanism that underlies the effect of homophily strength on the likelihood of bi-polarization. The longer it takes before the equilibrium of bi-polarization is reached, the more likely it is that in the process agents interact with dissimilar others and learn arguments counter to their current opinion tendency. If this happens, it is likely that agents further spread the counter arguments within the subset of the population that leans towards the same pole of the opinion spectrum. As a consequence, bi-polarization declines again and the population becomes more likely to move towards the other possible equilibrium, perfect consensus. This explains why perfect group splits occur only rarely under moderate homophily [55].

To summarize, our computational experiments yielded two main findings. First, the informal reasoning proposed above is consistent: The interplay of homophily and argument communication can entail bi-polarization. Second, bi-polarization obtains only when homophily is sufficiently strong. To assure that these conclusions can be generalized beyond the specific parameter setting that we inspected in the computational experiments reported in this paper, we have conducted extensive additional tests, varying the remaining parameters of our model (*N*, *P*, *C*, *S*). We have not found any combination of these parameters that generated a stable split into two maximally dissimilar subgroups under weak homophily (*h*<2). This suggests that strong homophily is a necessary condition of bi-polarization.

### 1.4. The Empirical Study

#### 1.4.1. Overview

The purpose of the laboratory experiment was twofold. First, we aimed to test the new theoretical element that ACTB adds to existing models of social influence, the communication of arguments. More specifically, we tested in a computerized social-influence experiment with human subjects, whether social influence in terms of argument communication results in opinion dynamics that differ in the theoretically predicted way from those dynamics that communication in terms of opinions creates. To this end, we compared empirical opinion dynamics in three between-subjects treatments. In the *Only-opinion-condition*, participants informed each other only about their current opinion without adding any further information. This treatment was designed to test the influence process that is assumed both in classical models of positive social influence, as well as in models that combined positive and negative influence on continuous opinions, where agents change their opinions directly in response to observed opinions of other agents [17], [22]–[25]. The second condition of our experiment was the *Only-argument-condition*. In this condition, subjects could transmit only arguments pro or con a particular position on the issue at stake during the discussion, but did not observe others’ opinions. This condition allowed to test the influence process that ACTB assumes. We test in our experiment each of the models under the scope-condition that matches their theoretical assumptions about the influence process. This does, however, give no insight into which models’ predictions give a better match with the empirically observed pattern when both types of influence processes can occur simultaneously. Accordingly, we included into our experiment a third treatment where participants transmitted both their opinion and an argument (*Opinion-and-arguments-condition*). This allows to test whether the effects of argument communication are robust to the presence of direct influence from exposure to others’ opinions.

The second purpose of the empirical study was to test whether communication of arguments can result in bi-polarization, putting to the test the core prediction of ACTB. This requires an experimental design that allows drawing conclusions about whether observed bi-polarization tendencies have been caused by argument communication or by the combination of positive and negative influence. To this end, we took advantage of a result of our computer simulation experiment, namely that ACTB predicts bi-polarization to obtain only when individuals are exposed to interaction partners with similar opinions (homophily). As we will show in detail below, social-influence models that assume only positive influence as well as models that combine positive with negative influence do not generate bi-polarization under this condition. In contrast, models that include negative influence predict bi-polarization to obtain when individuals with dissimilar opinions interact. Therefore, participants of all three experimental treatments interacted in the first part of the experiment with others who held similar opinions (homophilous matching of interaction partners). Subsequently, participants with dissimilar opinions where matched for interaction (heterophilous matching).

To be sure, the experiment was primarily designed to test ACTB and should not be understood as a general test of the negative-influence assumption. Our design does inform about whether the bi-polarization tendencies that we observed in this experiment were the result of argument communication or negative influence. However, not finding support for negative influence in this experiment does not challenge this assumption in general as it is still possible that negative influence plays an important role in other contexts. For instance, it has been argued that individuals are negatively influenced by interaction partners who differ on socio-demographic dimensions or are perceived to belong to an out-group because these differences may entail disliking [2], [14], [28], [91]. In the experiment, however, we decided not to inform participants about socio-demographic characteristics or group-memberships of their interaction partners, deliberately making disliking and negative influence unlikely from the perspective of those theories. If we find bi-polarization in this setting, this would be surprising from the point of view of earlier explanations, because it would show that bi-polarization can emerge even in the absence of negative influence.

The experimental data will be provided by the first author upon request.

#### 1.4.2. Experimental design

In each experimental session, we invited 8 participants to a computer laboratory where they sat in separate cubicles. We informed them that they would not be deceived during the experiment and that we had designed the experiment in order to study the formation of individual opinions in a social setting. Participants were asked to imagine that they were member of a discussion group that talks about the best location for building a new leisure center. This new center could be constructed in one of two hypothetical towns (town A and town B) or at any place in between these two towns. We chose this artificial issue because participants had no previous knowledge about it. This made it possible to impose the arguments that were known to each of the participants. In addition, the best spot for the leisure center can be identified on an interval scale, providing the participants an unambiguous way to inform each other about their opinion. After all participants had confirmed that they had understood the instructions, we started the computer program that ran the experiment. From this moment on, communication took place on the computers screens.

In the first phase of the experiment, each participant received a different set of three arguments. Each argument suggests that either town A or town B is the better place for the new leisure center. For example, one of the pro town A arguments reads: “There is a university in town A. The nearer the leisure center will be build to town A, the more students will be attracted”. Altogether there were six arguments pro town A and six pro town B. Half of the participants received two arguments pro town A and one pro town B and the other half received one pro town A and two pro town B. In the following, we therefore refer to those participants who received two pro town A arguments as “A-types” and to the others as “B-types”. Whether a specific participant was of type A or B was assigned randomly. In pilot studies, we asked participants to rate the importance of 20 arguments on a seven-point scale ranging from “very unimportant” (1) to “very important” (7). In the experiment, we included only those 12 arguments with an average rating of at least 5.5.

After each participant had read the initial set of arguments, participants expressed the first time their opinion about the best location for the new leisure center. We used a scale ranging from −50 (*town A*) to +50 (*town B*). Participants could choose any value between the two extremes.

In the second phase, each participant interacted once with each of the seven other participants of the session. In the first three rounds (homophilous matching phase) interactions took only place between participants who had received the same number of pro town A and pro town B arguments, imposing homophily, the central precondition of bi-polarization according to the new theory. In the remaining 4 interactions, participants were subsequently matched with the 4 participants of their session who had another number of pro-A and pro-B arguments (heterophilous matching phase). We used this interaction schedule in all three between-subject conditions. Participants were not aware of the schedule. We only informed them that they would interact once with each participant of the experiment. All interactions did really take place. Participants were not deceived.

The experiment focused on testing whether the communication of arguments implies bi-polarization and was designed to control as much as possible for effects of other model ingredients. In particular, we sought to prevent that in case of falsification of our predictions, other model ingredients could be held responsible for the lack of support for the new model. One possible reason for falsification could be that in the experiment, with its relatively short duration in time, individuals might not forget arguments that they learned in previous interactions. We even deliberately suppressed this possibility in the experiment in order to prevent that selective remembering of arguments could blur the link between arguments and opinion. Participants could always read a complete list of those arguments which they had received at the very beginning and which they had come across during the experiment. If individuals do not forget arguments, however, then repeated argument exchange will result in a situation where all participants consider relevant all existing arguments for opinion formation and, in turn, adopt a moderate opinion. In other words, if participants of the experiment do not forget arguments, then bi-polarization would be rather unlikely. To allow bi-polarization while precluding forgetting at the same time, we implemented that all participants who received two pro town A (B) arguments received the same pro town B (A) argument. As a consequence, participants who received during the homophilous-matching phase an argument against their initial tendency, always received an argument that was already known to them before the exchange of arguments. Argument supporting the initial tendency, however, could be new to the participant. With this, it was possible that a participant’s initial tendency could intensify through interaction, but this was at the same time not guaranteed because opinions were not determined by the arguments.

We made participants explicitly aware of the fact that they had received different sets of arguments. However, we did not inform them about the exact distribution of arguments. Hence, participants were not aware of the two types and thus no social categorization was possible on basis of the initial distribution of arguments.

In the *Only-opinion-condition*, each interaction consisted of two steps. First, the computer informed the participants about their partners’ opinion on the best location for the leisure center, showing the partner’s most recent opinion rating. Second, all participants rated again where they personally thought the best place for the leisure center was. The interactions in the *Only-argument-condition* consisted of three steps. First, both interaction partners were asked to select which of their arguments should be transmitted to their current interaction partner. Second, participants read which argument their respective partner had transmitted. Whenever a participant had received a new argument then this argument was added to this participant’s list of arguments and could later be transmitted to interaction partners. Finally, the participants expressed their opinion again. The new opinion rating, however, was not communicated to the current interaction partners. The *Opinions-and-arguments-condition* was very similar to the *Only-argument-condition* except for the fact that in step 2, participants did not only read the transmitted argument but also learned the opinion of the respective partner about the best location of the leisure center.

### 1.4.3. Predictions of the Competing Models of Social Influence

Models of social influence predict critically different opinion dynamics to obtain in the setting of the experiment. We focus here on three models of social influence: (i) standard social influence models which assume only positive influence, (ii) models that also include negative influence, and (iii) ACTB. In order to derive exact predictions about the opinion dynamic that each of these three theories implies in the setting of the laboratory experiment, we included the main design features of the experiment in the formal models.

First, we included assumptions about the initial opinions of the participants, which followed from the initial assignment of arguments in the experiment. Thus, we implemented that four participants of each discussion group should hold negative opinion values (A-types) and that the remaining opinions should be positive (B-types). To be more precise, equation 1 implies that participants held opinions of −16.66 or +16.66. These values represent opinions on the opinion scale that was used in the experiment which ranges from −50 (*town A*) to +50 (*town B*). In order to use the same opinion scale as existing models and ACTB, we linearly transformed the opinions to a value range between −1 and +1, arriving at initial opinion values of −.33 (A-types) and +.33 (B-types). Second, we implemented the interaction schedule that we imposed in the experiment (first homophilous then heterophilous matching).

#### Standard social-influence models

These models operationalize social influence as averaging, an assumption that we implemented with Equation 4. This equation implies that the opinion *o_i,t_* of agent *i* at interaction period *t* is a function of her previous opinion (*o_i,t−1_*) and the opinion of the interaction partner (*o_j,t−1_*). The influence weights *w_ij,t_* describe the direction and strength of influence that participant *j* exerts influence on *i*’s opinion and were fixed to the value of +1 for all pairs of interaction partners. This assumes that influence is always positive. Equation 4 furthermore implies that the relative impact of the interaction partner’s opinion declines in the number of previous interactions of the focal agent, which implements the assumption that agents consider the opinion of all previous interaction partners with equal weight when opinions are updated.
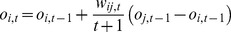
(4)


**Figure 4:**
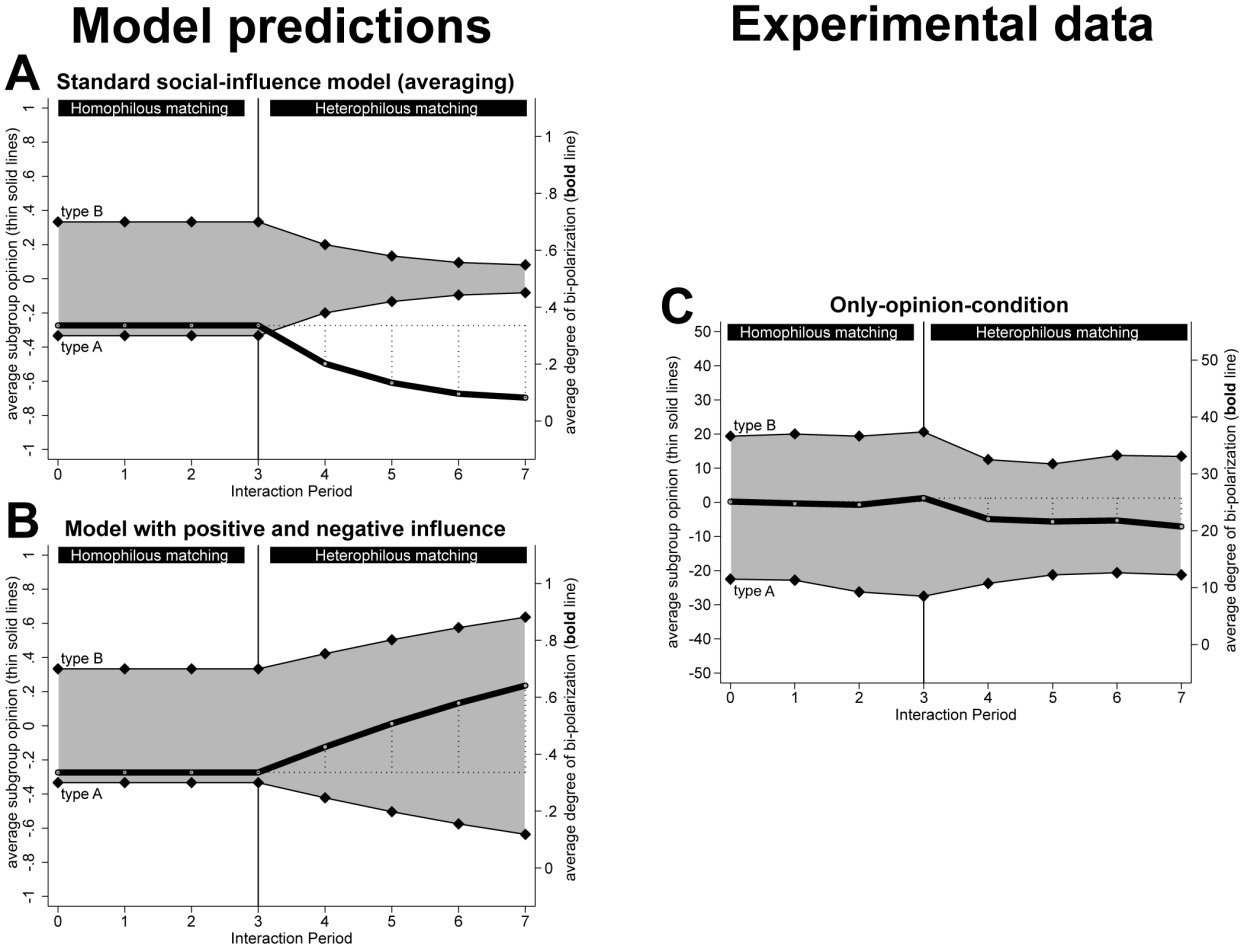
Predictions of existing social-influence models (left panel) and results of the experiment (right panel).

In Figure 4, Panel A shows the opinion dynamics, which follow deterministically from equation 4 and the assumptions about the initial opinions and the interaction schedule that we implemented in the experiment. The upper (lower) thin solid line depicts the average opinion of the 4 participants of type B (A). The distance between the two lines (highlighted by the gray area) serves as a measure of opinion distance between the two types. The bold lines inform about the degree of bi-polarization, which was measured in the same way as in the computational experiment reported above and informs about the degree to which the groups fall apart into homogeneous subgroups with opposed opinions. The dotted lines in the graphs highlight the changes in average degree of bi-polarization during the first three (homophilous matching) and the last four interactions (heterophilous matching).

During the first three interactions, participants interacted with those group members who held an identical opinion. The averaging assumption that is operationalized in Equation 4 implies that these interactions do not result in opinion changes. Accordingly, the graph in Panel A of Figure 4 shows that according to classical social-influence models neither the degree of bi-polarization nor the opinion averages of the two types of discussion members should change during interaction periods 1 to 3. In the remaining periods, however, participants were matched with those group members who held dissimilar opinions. According to classical models of social influence, this should motivate participants to develop more moderate opinions. As the graph shows, opinions should then converge and the degree of bi-polarization should decrease during the final four interaction periods.

#### Models combining positive and negative influence

These models assume that individuals are influenced positively by interaction partners with similar opinions, and that influence turns negative when individuals with dissimilar opinions interact. Also this model was implemented with Equation 4, but with additional assumptions about the influence weights which are summarized in Equation 5 (which we adapted from Flache and Mäs 2008a).
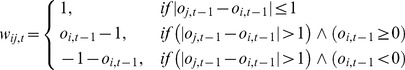
(5)


Equation 5 implies that influence weights adopt a value of +1, whenever the opinions of two interaction partners do not differ more than half of the range of the opinion scale. This implements positive influence by similar interaction partners. However, weights adopt negative values and result in negative influence when opinion differences between interaction partners exceed the threshold of half of the range of the opinion scale. Equation 5 assures that opinions remain within the bounds of the opinion scale (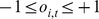). To this end, negative influence weights adopt smaller absolute values when *i* already holds an extreme opinion.

The model with both positive and negative influence generates predictions for the only-opinion condition in our experiment. These predictions follow deterministically from Equations 4 and 5 and are graphically summarized in Panel B of Figure 4. The graph shows that opinions remain unchanged when interaction partners hold identical opinions (homophilous matching). However, negative influence results in more extreme opinions and bi-polarization when dissimilar participants interact (heterophilous matching).

#### Predictions of ACTB

In order to implement the design elements of the only-argument condition of our experiment as closely as possible in the formal model, we adjusted those model assumptions that concern the forgetting of arguments. Participants could always see a complete list of those arguments that they had received at the very beginning of the experiment plus those arguments that they had received from interaction partners. This was included to standardize the sending and receiving of arguments, making sure that participants send exactly the same arguments that they had received earlier. At the same time, this procedure assured that participants would not forget arguments. To include this in the formal model, we assumed that agents consider all arguments as relevant that they came across at least once during the influence process. Technically, this implies that the number of salient arguments (*S_i,t_*) can vary between agents and increase over time.

**Figure 5:**
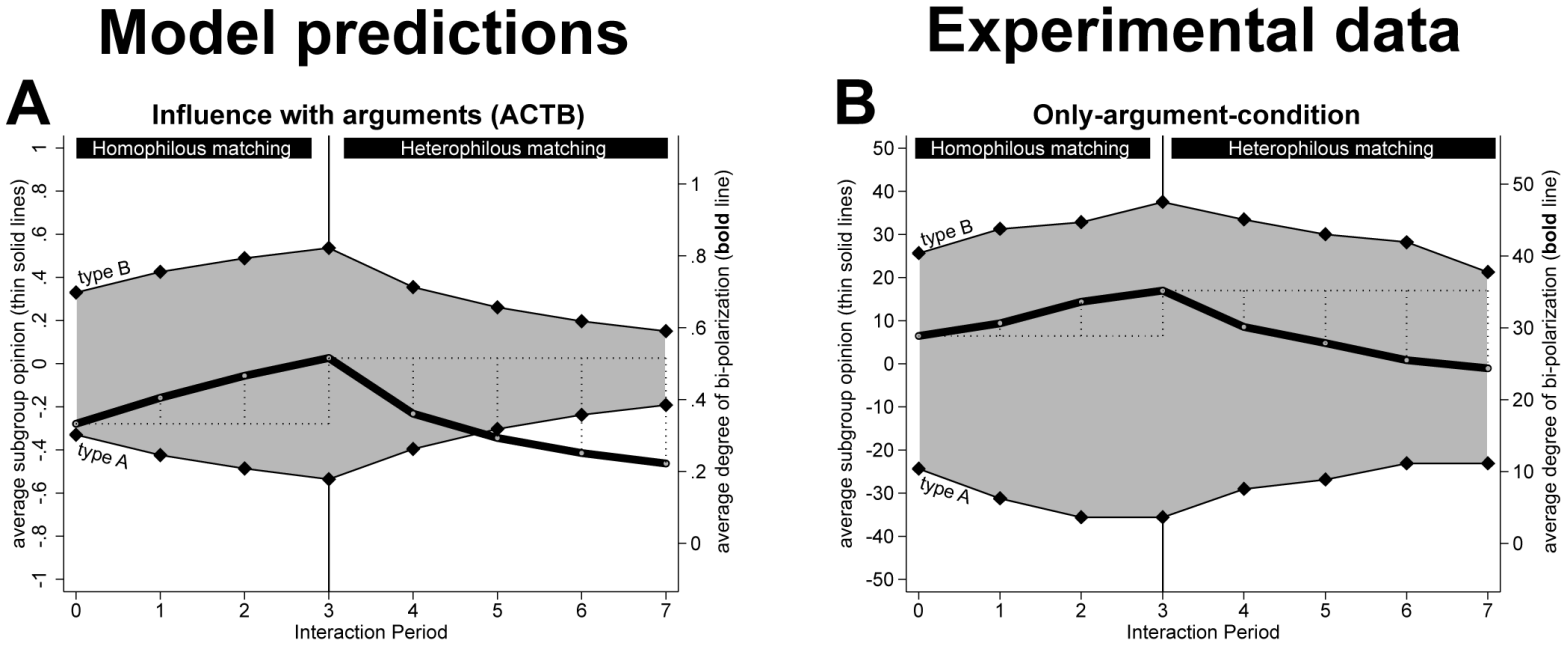
Predictions of ACTB (left panel) and results of the experiment (right panel).

Finally, we implemented the same interaction schedule and the same initial assignment of arguments as in the laboratory experiment (*P* = *C* = 6; *S_i,_*_1_ = 3). Unlike the models for the only- opinion condition, the formal model contains even with these modifications still a random component in the selection of the communicated argument. Therefore, we conducted 500 independent replications of the social-influence process and report average developments in Figure 5.

Panel A in Figure 5 shows that the exchange of arguments with agents who hold similar opinions leads according to ACTB to intensified opinions and bi-polarization during the first three interaction periods. This is because interaction partners either provide each other with a new argument that supports their initial opinion or with a counter argument that they already know. However, as the results for the heterophilous interaction phase show, ACTB predicts that argument exchange with the remaining agents (periods 4 thru 7) leads to more moderate opinions as agents are mainly exposed to arguments that do not support their current opinions.

#### Comparison of the predictions

Panel A and B of Figure 4 and Panel A of Figure 5 visualize that the three formal models of social influence imply critically different predictions for the setting of the experiment. Two main differences draw attention. First, only the model which includes positive and negative influence (Panel B in Figure 4) predicts bi-polarization during the heterophilous matching phase. Hence, observing this dynamic under the experimental conditions where participants inform each other about their opinions would support that interaction with dissimilar others leads to negative influence. The model with positive and negative influence is not directly applicable to the *Only-argument-condition*, as participants were not informed about the opinion of their current interaction partners. However, it can not be fully precluded that participants inferred the opinion of their interaction partner from the arguments they transmitted. Thus, increasing degrees of bi-polarization during heterophilous matching in the *Only-arguments-condition* would also support the negative influence model.

Second, Panel A of Figure 5 shows that only ACTB predicts bi-polarization during the homophilous matching phase. Finding this dynamic in the conditions where participants could exchange arguments would, thus, support ACTB. As participants in the *Only-opinion-condition* could not communicate arguments, finding bi-polarization during the homophilous matching phase of this condition should not be interpreted as support for ACTB.

#### 1.4.4. Participants

Members of a general pool of participants at the Department of Sociology at the University of Groningen had been invited to participate in this experiment. Interested students could register for a specific session using an online form [92]. The study has been approved by the review board of the Department of Sociology at the University of Groningen. Written informed consent was obtained from each participant before conducting the experiment. The recruitment and the experiment complied with the guidelines set out by the Sociological Laboratory of the Department of Sociology at the University of Groningen (http://www.gmw.rug.nl/~orsee/public/index.php?language=en).

We assigned the sessions randomly to the three experimental conditions. Participants received monetary compensation. After excluding problematic sessions (see below), we included data of 65 female and 31 male participants in the analyses (N = 96). On average, participants were 23 years old.

Altogether, we conducted 18 sessions with 8 participants per session. However, we excluded 6 sessions from the analysis because the manipulation of the initial opinions did not work out. Even though participants received at the beginning of the experiment two arguments favoring one of the two towns, it was still possible that participants considered the one argument in favor of the other town as most relevant. In some cases, participants’ initial opinion therefore tended towards the town for which fewer arguments were given. All sessions in which this happened for more than one of the participant were excluded from the analysis. This was necessary to ensure that the interaction schedule imposed homophilous matching during the first three interactions. Altogether, we used data from twelve sessions with eight participants each for the statistical analyses (N = 96). For each of the three conditions, four sessions are available (N = 32 each).

#### 1.4.5. Results

**Figure 6:**
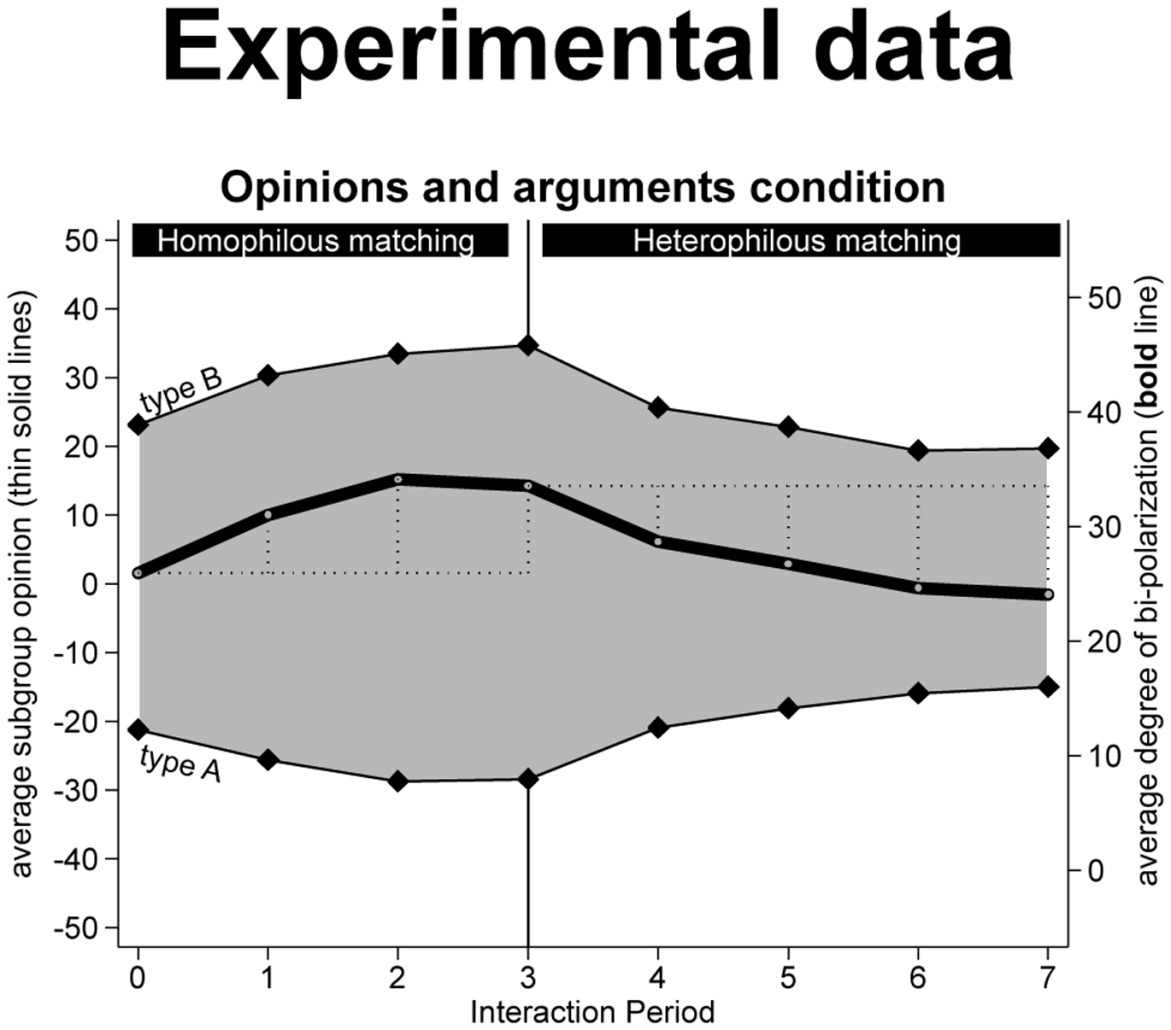
Dynamics found under the Opinions-and-arguments-condition.

Panel C in Figure 4, Panel B in Figure 5, and Figure 6 picture the observed bi-polarization dynamics in the three conditions of the experiment. Results are visualized in the same way as the theoretical predictions. This allows direct comparison of the predictions of the three theoretical models (panels on the left-hand side) with the empirical findings for the corresponding conditions of the experiment (panels on the right-hand side). To also quantify bi-polarization dynamics in the three experimental conditions, we estimated for each condition separately a linear regression with the degree of bi-polarization in each experimental session as dependent and two period effects as independent variables. The first period effect is designed to capture a possible change in the degree of bi-polarization during the first three interactions (Periods are coded: 0 1 2 3 3 3 3 3) and the second allows to assess how much the degree of bi-polarization changed in the remaining 4 interactions (Periods are coded: 0 0 0 0 1 2 3 4). The regression coefficients of the two period effects indicate whether there was bi-polarization (positive coefficient); no change (insignificant coefficient); or whether opinion distance between the two types decreased (negative coefficient). For each condition there were 32 observations available (4 sessions×8 opinion measurements). In the following, we report the results of these regression analyses. In addition, the estimates are summarized in Table S1 of the supplementary information.

The figures show for all three conditions that there were significant differences between the opinion averages of the two types of participants already before the first interaction (interaction period = 0). Also the initial degree of bi-polarization was in all conditions significantly different from zero (t-values of intercepts in the regressions range from 15.03 to 24.43). This demonstrates that the assignment of arguments led to the desired opinion differences between the two types. Comparing the initial degree of bi-polarization which we observed in the experiment (right panel) with the expected initial degree of bi-polarization (left panel), one can see that the observed values even exceed the expectations. This discrepancy does not affect our test, however, because we focus on the direction of change of opinion differences rather than the absolute opinion differences.

In the *Only-opinion-condition* of the experiment, the degree of bi-polarization hardly changed during the first three interactions. Actually, it decreased on average by 0.21 during the first three interactions. The decrease is not significantly different from zero (t = −0.42). This was different under the *Only-argument-condition* and the *Opinions and arguments-condition*. In both conditions the degree of bi-polarization significantly increased per interaction, by 1.71 (t = 2.98) and 1.91 (t = 2.15) respectively.

Dynamics changed when interaction partners with different opinions were matched (interaction period>3). Under all three conditions of the experiment, we found decreasing opinion differences between the two types of participants. In the *Only-opinion-condition*, the degree of bi-polarization decreased on average by 1.01. This effect differs significantly from zero (t = −2.77) but the confidence interval of the effect reveals that it does not differ significantly from the weak decrease during the first three interactions. In the *Only-argument-condition* and the *Opinions and arguments-condition*, the degree of bi-polarization decreased from interaction period 4 on by 2.75 (t = −6.5) and 2.74 (t = −4.17) on average. In both conditions, this decrease during the interactions 4 thru 7 differs significantly from zero and therefore also from the *in*crease during the first three interactions.

We also wanted to test whether or not the dynamics of bi-polarization differed significantly between the three conditions. To this end, we estimated a regression that tested differences between the *Only-opinion-condition* (reference category) on the one hand and the *Only-argument-condition* and the *Opinions and arguments-condition* on the other hand. For this purpose, we used the same regression approach as for the separate models and included main and interaction effects for the experimental conditions. The results are summarized in Tables S2 and S3. It turned out that the increase in the degree of bi-polarization during the first three interactions was significantly stronger in both the *Only-argument-condition* (t = 2.01) and the *Opinions and arguments-condition* (t = 2.22) than it was in the *Only-opinion-condition*.

We found furthermore that the decrease in the degree of bi-polarization during the final four interaction periods was significantly stronger in the *Only-argument-condition* (t = −2.47) and the *Opinions and arguments-condition* (t = −2.44) as compared to the *Only-opinion-condition*. A comparison of the differences between the *Only-argument-condition* and the *Opinions and arguments-condition* revealed that both the developments during the first three interactions (t = .19) and the subsequent finteractions (t = .02) did not differ significantly between the two conditions.

In sum, there are three main findings. First, we found opinion convergence during the heterophilous-matching phase of all experimental conditions. This challenges the prediction of social-influence models that assume both positive and negative influence (see Panel B of Figure 4) and demonstrates that in the setting of this experiment participants were not negatively influenced by their interaction partners.

Second, in both conditions where participants transmitted arguments we found bi-polarization during the homophilous matching phase. This supports ACTB because only ACTB is able to explain bi-polarization in this phase of the experiment. What is more, bi-polarization was not found during the homophilous matching phase under the *only-opinions-condition*, suggesting that the core theoretical ingredient that ACTB adds to existing models of social influence, argument communication, is responsible for the bi-polarization tendencies which we found under the conditions where participants transmitted arguments.

Third, we did not find significant differences in the bi-polarization dynamics under the *Only-argument-condition* and the *Opinion-and-argument-condition*. This indicates that the effect of argument exchange is robust to influences of opinions.

## Conclusions

In this article, we have provided both theoretical and empirical support for the claim that bi-polarization can emerge even when individuals do not seek to increase opinion differences to disliked members of the population. Our theoretical contribution focused on demonstrating the logical validity of this claim, presenting and analyzing a formal model of social influence that includes argument communication, the core ingredient of the new theory.

Our laboratory experiment supported that even in a setting where we did not find support for negative influence groups may exhibit bi-polarization. What is more, we found bi-polarization only in those experimental conditions where social influence was based on argument communication and did not find increasing opinion differences in a condition where social influence was based only on opinions. This supports our claim that communication based on arguments implies substantially different opinion dynamics than communication that is based on opinions. What is more, experimental results suggest that the bi-polarization tendencies that are created by the communication of arguments are robust to the effects of simultaneous opinion communication. In sum, the results of our experiments support that argument communication in tandem with homophily can give rise to bi-polarization even in the absence of negative influence based on exposure to opinions.

These findings provide the following insights for future research. First, future modeling research is needed to compare ACTB with an alternative approach that was recently proposed by Dandekar et al. [93]. This model assumes so-called “biased assimilation”, the tendency of individuals to readily accept evidence that confirms their opinions while carefully scrutinizing disconfirming information. Unlike in our formal representation of ACTB, information sharing and processing is not modeled explicitly by Dandekar et al. However, it is assumed that individuals’ opinions intensify when they interact with likeminded others, which is a core implication of ACTB. Therefore, future research should explore to what extent the two models predict bi-polarization under similar conditions.

Second, our study explored whether the interplay of homophily and argument exchange can lead to bi-polarization, testing the possibility to explain bi-polarization of continuous opinions without assuming negative influence. Nevertheless, an open question is whether empirical bi-polarization tendencies [33]–[37], [94] are better explained with negative influence, or with argument communication. Answering this question is an intricate problem because the two mechanisms are very similar in their predicted outcomes and might also act in tandem. It appears possible, for instance, that the interplay of strong homophily and argument exchange gives rise to opinion differences in an initially homogenous population. Once these differences have become sufficiently pronounced and salient, negative influence might unfold and further intensify them.

A potential strategy to disentangle the effects of argument exchange and negative influence and to assess which mechanism dominates under given conditions might be to study effects of the so called “timing of contacts” [28]. Our experimental results support that influence with persuasive arguments increases bi-polarization when individuals interact in a first phase with similar others and leads to opinion convergence when individuals with opposing views are brought into contact in a second phase. Strikingly, models that assume negative influence predict the opposite dynamic [28]. In the first phase, similarity between interaction partners results in their model in converging views. In the second phase, however, opinion differences create negative influence and bi-polarization. Hence, experimentally manipulating the timing of contacts might allow testing whether bi-polarization tendencies result from negative influence or homophilous argument exchange.

Third, our computer-simulation study found that the exchange of persuasive arguments entails bi-polarization only if the selection of interaction partners is shaped by strong homophily, suggesting that a moderate tendency to interact with likeminded others may not suffice to create bi-polarization as long as individuals occasionally engage in exchange of arguments with people who hold dissimilar opinions. Empirical research shows that homophily is a strong force in many social settings [51]. However, it appears questionable whether homophily is strong enough to explain e.g. the increasing divide on issues like abortion, sexual morality and the war in Iraq that scholars have observed in contemporary America [15], [34], [38], [94].

On the other hand, focusing on the core mechanisms that might create bi-polarization, our formal model abstracts from parallel processes that may amplify the bi-polarizing effects of argument exchange. For instance, trying to avoid cognitive inconsistency [58], [59], individuals may tend to systematically disregard those arguments that contradict their current opinion, a process that is similar to the notion of biased assimilation as studied by Dandekar et al. [93]. Accordingly, individuals may tend to base their opinions only on arguments that support their opinions and may hesitate to bring up counter arguments during argument exchange with likeminded others. This process has the potential to amplify the bi-polarization tendencies that argument communication and homophily cause in our model and might therefore help explaining bi-polarization in settings where homophily is weak.

What is more, observers of the internet have raised concerns that recent technical developments of social networking sites and internet search-engines create an additional source of homophily which may be neither wanted nor recognized by users but has the potential to dramatically shape interaction [93], [95], [96]. New linking algorithms on social networking sites establish network contacts between users with similar interests and systemically cut off ties between users who do not interact frequently. Users complain that their social networks on these sites have turned very homogeneous and consist nearly exclusively of likeminded friends even though they used to have links to users with opposite political attitudes [95]. A similar effect has been attributed to Internet search engines that use personalized search algorithms. These algorithms have been developed to generate search results that reflect the interests of individual users. A disadvantage, however, may be that internet users will find it difficult to locate websites which support opposing opinions. In sum, these new technologies increase the degree to which users are exposed to arguments of likeminded others, making bi-polarization more likely according to ACTB. Future empirical research and modeling work is needed to understand the impact of these new technologies on the social networks of Internet users and their potential impact on large-scale opinion dynamics.

## Supporting Information

Table S1 Bi-polarization dynamics in the 3 conditions of the experiment.
(DOCX)

Table S2 Comparison of bi-polarization dynamics (*only-opinions-condition* vs. *only-arguments-condition* and *opinions and arguments-condition*).
(DOCX)

Table S3 Comparison of bi-polarization dynamics (*only-arguments-condition* vs. *opinions and arguments-condition*).
(DOCX)

Supporting Information S1 (DOCX)
