# Supplementary material for: Differentiation without Distancing. Explaining Bi-Polarization of Opinions without Negative Influence
Source: PLoS One. 2013 Nov 27;8(11):e74516. doi: 10.1371/journal.pone.0074516 (PMC3842239; doi:10.1371/journal.pone.0074516)
Supplement: Table S1 — Bi-polarization dynamics in the 3 conditions of the experiment. (DOCX) [file pone.0074516.s001.docx]

**Table S1. Bi-polarization dynamics in the 3 conditions of the experiment**

|  | Only-opinions-condition | Only-arguments-condition | Opinions and arguments |
| --- | --- | --- | --- |
| constant | 25.054 | 29.104 | 27.714 |
|  | (24.29)** | (24.43)** | (15.03)** |
| Periods 1-3, | -0.210 | 1.715 | 1.913 |
| (homop. matching) | (0.42) | (2.98)** | (2.15)* |
| Periods 4-7 | -1.016 | -2.758 | -2.739 |
| (heterop. matching) | (2.77)** | (6.50)** | (4.17)** |
| *R*^2^ | 0.32 | 0.60 | 0.38 |
| *N* | 32 | 32 | 32 |

t-values in parentheses; * *p*<0.05; ** *p*<0.01
