# Supplementary material for: Differentiation without Distancing. Explaining Bi-Polarization of Opinions without Negative Influence
Source: PLoS One. 2013 Nov 27;8(11):e74516. doi: 10.1371/journal.pone.0074516 (PMC3842239; doi:10.1371/journal.pone.0074516)
Supplement: Table S2 — Comparison of bi-polarization dynamics (only-opinions-condition vs. only-arguments-condition and opinions and arguments-condition). (DOCX) [file pone.0074516.s002.docx]

**Table S2: Comparison of bi-polarization dynamics (*only-opinions-condition* vs. *only-arguments-condition* and *opinions and arguments-condition*)**

| *Reference category: only-opinion-condition* | |
| --- | --- |
| constant | 25.054 |
|  | (17.89)** |
| Periods 1-3 | -0.210 |
|  | (0.31) |
| Periods 4-7 | -1.016 |
|  | (2.04)* |
| *Comparison with only-arguments-condition* | |
| Condition dummy | 4.050 |
|  | (2.05)* |
| Periods 1-3 × dummy | 1.925 |
|  | (2.01)* |
| Periods 4-7 × dummy | -1.742 |
|  | (2.47)* |
| *Comparison with opinions and arguments-condition* | |
| Condition dummy | 2.660 |
|  | (1.34) |
| Periods 1-3 × dummy | 2.123 |
|  | (2.22)* |
| Periods 4-7 × dummy | -1.723 |
|  | (2.44)* |
| *R*^2^ | 0.60 |
| *N* | 96 |

t-values in parentheses; * *p*<0.05; ** *p*<0.01
