# Supplementary material for: Differentiation without Distancing. Explaining Bi-Polarization of Opinions without Negative Influence
Source: PLoS One. 2013 Nov 27;8(11):e74516. doi: 10.1371/journal.pone.0074516 (PMC3842239; doi:10.1371/journal.pone.0074516)
Supplement: Table S3 — Comparison of bi-polarization dynamics (only-arguments-condition vs. opinions and arguments-condition). (DOCX) [file pone.0074516.s003.docx]

**Table S3: Comparison of bi-polarization dynamics (*only-arguments-condition* vs. *opinions and arguments-condition*)**

| *Reference category: only-arguments-condition* | |
| --- | --- |
| constant | 29.104 |
|  | (18.75)** |
| Periods 1-3 | 1.715 |
|  | (2.29)* |
| Periods 4-7 | -2.758 |
|  | (4.99)** |
| *Comparison with opinions and arguments-condition* | |
| Condition dummy | -1.390 |
|  | (0.63) |
| Periods 1-3 × dummy | 0.198 |
|  | (0.19) |
| Periods 4-7 × dummy | 0.019 |
|  | (0.02) |
| *R*^2^ | 0.47 |
| *N* | 64 |

t-values in parentheses; * *p*<0.05; ** *p*<0.01
