# Supplementary material for: Differentiation without Distancing. Explaining Bi-Polarization of Opinions without Negative Influence
Source: PLoS One. 2013 Nov 27;8(11):e74516. doi: 10.1371/journal.pone.0074516 (PMC3842239; doi:10.1371/journal.pone.0074516)
Supplement: Supporting Information S1 — (DOCX) [file pone.0074516.s004.docx]

# Supporting information

**Differentiation without distancing. Explaining bi-polarization without negative influence**

## Description of ideal-typical runs shown in Figure 1 of the paper

The purpose of Figure 1 of the main paper is to provide an overview over the central contributions to the literature on formal models of continuous opinion dynamics by illustrating opinion dynamics that existing models of social influence generate. Panel A shows that classical models of social influence, which assume that influence is always positive, imply opinion convergence and the emergence of a perfect opinion consensus in the long run. Models that add the mechanism of homophily (see Panel B) predict the development and persistence of several clusters with relatively moderate opinions. However, Panel C shows that this prediction is not robust to interaction noise. That is, opinion clustering breaks down when there is a very small but positive probability that members of distinct clusters influence each other. Panel D focuses on models that include negative influence. These models assume that influence is positive only when agents hold similar opinions and turns negative when agents hold very distinct views. Panel D shows that this class of models is able to generate bi-polarization of opinions.

In order to generate the four graphs of Figure 1, we implemented the core assumptions of existing models in our own simulation software. In the following, we summarize the models and the parameter values that we assumed when we generated the ideal-typical simulation runs. For detailed analyses of the models, we refer to the original publications.

We assumed for all simulations that the population consists of 100 agents (*N*=100), and that each agent *i* is described by one opinion value *o_i,t_*, which is open to influence. Existing models differ with regard to the assumed range of the opinion scale. Classical social influence models, for instance, typically assume a scale that ranges from zero to one ($0\leq o_{i,t}\leq1$). Many models that include negative influence [[e.g. 1](#_ENREF_1)], in contrast, assume opinions that can adopt values between -1 and 1 ($-1\leq o_{i,t}\leq1$). All four simulation runs in Figure 1 were conducted with an opinion scale that ranged from zero to one ($0\leq o_{i,t}\leq1$). However, in order to be consistent with the opinion scale used in the ACTB model (see Figure 2 of the main paper), we linearly transformed the simulated opinions to a scale that ranges from -1 to 1 ($-1\leq o_{i,t}\leq1$) when we prepared Figure 1.

In all simulation runs that we show in Figure 1, initial opinion values *o_i,_*_0_ were randomly assigned (drawn from a uniform distribution). Furthermore, opinion dynamics were broken down to a sequence of simulation events. At each event *t*, the computer program randomly picked one agent *i* and updated this agent’s opinion *o_i,t_* in such a way that the new opinion $o_{i,t+1}=o_{i,t}+\Delta o_{i,t}$.

*Panel A: only positive influence.* This simulation run illustrates typical opinion dynamics that obtain when agents influence each other positively. To be more precise, in this simulation run we assumed that each agent shifts his opinion towards the weighted opinion average of the population, the standard operationalization of positive social-influence in models of continuous opinion dynamics [[2](#_ENREF_2),[3](#_ENREF_3),[4](#_ENREF_4),[5](#_ENREF_5),[6](#_ENREF_6)]. Technically^[[1]](#footnote-1)^,

$\Delta o_{i,t}=\frac{\sum_{j=1}^{N} \left( o_{j,t}-o_{i,t} \right)w_{ij,t}}{\sum_{j=1}^{N} w_{ij,t}}$. (1)

The influence weights $w_{ij,t}$ represent the degree to which agent *j* influences the opinion of agent *i*. Weights were assumed to be positive always, implementing that social influence is positive. Furthermore, we included that influence is stronger when agents hold similar opinions, which was integrated with Equation 2. For graphical reasons^[[2]](#footnote-2)^, we assigned the value 4 to parameter *a*.

$w_{ij,t}=\left( 1-\left| o_{j,t}-o_{i,t} \right| \right)^{a}$ (2)

*Panel B: positive influence, and homophily*. The opinion dynamics shown in Panel B were generated with the bounded-confidence model [[8](#_ENREF_8)], which implements that the opinion of the focal agent *i* is replaced by the average opinion of those agents that hold opinions within the focal agents’ confidence interval at the moment of updating ($o_{i,t}-\varepsilon\leq o_{j,t}\leq o_{i,t}+\varepsilon$). In other words, this model implements social influence in the same way as equation 1, but adds the assumption that influence weights *w_ij,t_* are either zero (when *i* and *j* disagree too much) or one (when *i* and *j* hold sufficiently similar opinions). For the simulation run in the figure, we assigned the value 0.2 to the confidence threshold $\varepsilon$.

*Panel C: positive influence, homophily, and interaction noise*. This panel is based on the same model and parameter values as Panel B, but we added interaction noise [[9](#_ENREF_9)]. That is, we included that there is a small probability of *p*=0.01 that an agent *j* exerts influence (*w_ij,t_*=1) on *i*’s opinion even though opinion distance between *i* and *j* exceeds the confidence threshold ($\left| o_{i,t}-o_{j,t} \right|>\varepsilon$).

*Panel D: positive, and negative influence.* The opinion dynamics shown in Panel D are based on Equation 1, but we included that influence weights $w_{ij,t}$ can adopt negative values, which incorporates negative influence [[1](#_ENREF_1),[7](#_ENREF_7)]. In particular, we assumed that influence is positive (*w_ij,t_*>0) when the opinion differences between *i* and *j* are smaller than a threshold of 0.5 (= 50% of range of opinion scale). If opinions differ more, then influence is negative (*w_ij,t_*<0). This was implemented with equations 3 and 4:

$w_{ij,t}=\left( 1-2\cdot\left| o_{j,t}-o_{i,t} \right| \right)$ if $\left| o_{j,t}-o_{i,t} \right|\leq0.5$ (3)

$w_{ij,t}=-1\left( 2\cdot\left| o_{j,t}-o_{i,t} \right|-1 \right)$ if $\left| o_{j,t}-o_{i,t} \right|>0.5$ (4)

References

1. Macy MW, Kitts J, Flache A, Benard S (2003) Polarization and Dynamic Networks. A Hopfield Model of Emergent Structure. In: Breiger R, Carley K, Pattison P, editors. Dynamic Social Network Modeling and Analysis: Workshop Summary and Papers. Washington, DC: The National Academies Press. pp. 162-173.

2. Abelson RP (1964) Mathematical Models of the Distribution of Attitudes Under Controversy. In: Frederiksen N, Gulliksen H, editors. Contributions to Mathematical Psychology. New York: Rinehart Winston. pp. 142-160.

3. Harary F (1959) A criterion for unanimity in French's theory of social power. In: Cartwright D, editor. Studies in social power. Ann Arbor: Institute for Social Research. pp. 168-182.

4. French JRP (1956) A Formal Theory of Social Power. Psychol Rev 63: 181-194.

5. Wagner C (1982) Allocation, Lehrer Models, and the Consensus of Probabilities. Theory and Decision 14: 207-220.

6. Berger RL (1981) A Necessary and Sufficient Condition for Reaching a Consensus Using DeGroot's Method. J Am Stat Assoc 76: 415-418.

7. Mäs M, Flache A, Kitts JA (in press) Cultural Integration and Differentiation in Groups and Organizations. In: Dignum V, Dignum F, Ferber J, Stratulat T, editors. Integrating Cultures: Formal Models and Agent-Based Simulations. New York: Springer.

8. Hegselmann R, Krause U (2002) Opinion Dynamics and Bounded Confidence Models, Analysis, and Simulation. JASSS-J Artif Soc S 5.

9. Mäs M, Flache A, Helbing D (2010) Individualization as Driving Force of Clustering Phenomena in Humans. PLoS Comput Biol 6: e1000959.

1. This model is analyzed in more detail in Mäs, Flache, and Kitts [[7](#_ENREF_7)]. [↑](#footnote-ref-1)
2. With this parameter value, opinion dynamics are not too quick that they can not be visualized. At the same time, dynamics are not too slow, preventing overly large figures. The value of *a* (*a*>1), does not affect the qualitative predictions of the model. [↑](#footnote-ref-2)
